# Mediterranean Diet Adherence and Nutritional Status in Dalmatian Kidney Transplant Recipients—Are They Related?

**DOI:** 10.3390/nu13093246

**Published:** 2021-09-18

**Authors:** Marijana Vučković, Josipa Radić, Andrea Gelemanović, Hrvoje Raos, Dora Bučan Nenadić, Ela Kolak, Mislav Radić

**Affiliations:** 1 Department of Nephrology and Dialysis, University Hospital Centre Split, 21000 Split, Croatia; mavuckovic@kbsplit.hr; 2 Department of Internal Medicine, University of Split School of Medicine, 21000 Split, Croatia; mislavradic@gmail.com; 3 Mediterranean Institute for Life Sciences (MedILS), 21000 Split, Croatia; andrea.gelemanovic@gmail.com; 4 Department of Family Medicine, Split-Dalmatia County Health Center, 21000 Split, Croatia; hrvoje.raos1995@gmail.com; 5 Department of Nutrition and Dietetics, University Hospital Centre Split, 21000 Split, Croatia; dorabucan@gmail.com (D.B.N.); elakolak93@gmail.com (E.K.); 6 Department of Clinical Immunology and Rheumatology, University Hospital Centre Split, 21000 Split, Croatia

**Keywords:** kidney transplantation, nutritional status, Mediterranean diet, body composition, nutrition, Dalmatian

## Abstract

The aim of this study was to evaluate adherence to Mediterranean diet (MeDi) and possible correlation of MeDi adherence and nutritional status parameters in Dalmatian kidney transplant recipients (KTRs). One hundred and sixteen KTRs were included in this study. Data about Mediterranean Diet Serving Score (MDSS), body mass composition, anthropometric parameters, clinical and laboratory parameters were collected for each study participant. The results showed 25% adherence to the MeDi in Dalmatian KTRs. MDSS showed association with higher serum albumin and phosphorus level and higher skeletal muscle mass. Also, significant association between diabetic status and MDSS was found. Adherence to olive oil intake suggested by the MeDi showed significant association with lower level of triglycerides and adherence to nuts suggestions was associated with lower level of fat mass. Following MeDi recommendations for consumption of other foods (cereals, potato, eggs, vegetables, fruits and dairy) were also associated with body mass composition parameters and laboratory findings. In conclusion, low adherence to the MeDi in Dalmatian KTRs raises high concerns. The results showed that MeDi can have favorable effects on nutritional status in KTRs. A structured nutritional approach is needed to enhance adherence to the MeDi and prevent possible adverse effects in this patient population.

## 1. Introduction

The Mediterranean diet (MeDi) has been recognized for its beneficial role in prevention and management of chronic diseases such as type 2 diabetes mellitus [1], hypertension [2], and metabolic syndrome [1]. MeDi is specifically is known for its effect on reduction of cardiovascular risk [3], and on greater lifespan (up to 2 years) in the general population [4].

Patients suffering from chronic kidney disease (CKD) require special care when it comes to nutrition, and it can be challenging to prescribe nutritional therapy to this group of patients. However, adherence to the MeDi has been associated with reduction of cardiovascular risk, lower mortality risk, lower risk of CKD progression and lower levels of oxidative stress in patients suffering from CKD [5].

Kidney transplantation (KTX) results in better quality of life and overall mortality, but these patients are still at high cardiometabolic risk, due to changes in nutritional status [6] and effects of polypharmacy following transplantation. Post-transplantation body composition modification is influenced by the effects of immunosuppressive therapy [7], level of physical activity, age and sex and it can affect overall cardiovascular risk and kidney function parameters in these patients [8,9].

Recent studies have stated that adherence to the MeDi can be associated with lower risk of kidney function decline and graft failure [10] as well as reduced risk of mortality and new onset diabetes after transplantation in kidney transplant recipients (KTRs) [11]. Besides that, MeDi has beneficial effects on oxidative stress levels [12], lipid levels [11,13] and incidence of metabolic syndrome [14] in KTRs.

The main aim of this cross-sectional study was to evaluate dietary habits and adherence to the MeDi of kidney transplant recipients from Mediterranean region of Dalmatia (Croatia). In addition, we examined the correlations of MeDi adherence with nutritional status, body composition and transplanted kidney function.

## 2. Materials and Methods

### 2.1. Study Design and Population

One hundred and fifty nine (159) kidney transplant recipients (KTRs) older than 18 years of age, with functioning kidney and no mobility difficulties, were included in this cross-sectional study conducted at the Outpatient clinic of the Department of Nephrology and Dialysis, University Hospital of Split, Croatia, between July 2019 and October 2019.

Some exclusion criteria were included and a written informed consent was obtained in all the participants. The study protocol was approved by the Ethics Committee of the University Hospital of Split.

### 2.2. Body Composition and Anthropometric Measurement

Body composition was assessed using an MC-780 Multi Frequency Segmental Body Analyzer (Tanita, Tokyo, Japan) for each study participant. The device uses a constant high frequency current flow and eight electrodes to determine electrical resistance of different tissues. The method is called bioelectrical impedance analysis (BIA). It is used to assess fat mass (kg), fat mass percentage (%), fat free mass (kg), visceral fat, muscle mass (kg), skeletal muscle mass (kg), skeletal muscle mass percentage (%) and body mass (kg). All patients were advised not to take any food or liquid at least 3 h before the measurement, to urinate just before the measurement and not to consume alcohol, eat or drink excessively nor to exercise in an excessive way at least one day before the body composition measurement [15]. Height was measured using a stadiometer. Waist circumference (WC) and mid-upper arm circumference (MUAC) were measured using a flexible, non-stretchable measuring tape in a standing position facing forward with their shoulders relaxed. Body mass index (BMI) and the waist-to-height ratio (WHtR) were calculated for each study participant.

### 2.3. Mediterranean Diet Serving Score

The Validated Mediterranean Diet Serving Score (MDSS) questionnaire was used to determine adherence to Mediterranean Diet (MeDi) considering the consumption of different foods and food groups (MeDi components) in time intervals per meal, day or week [16].

The food is divided into fourteen food groups, and points are given according to the new Mediterranean food pyramid in the following way: Three points for fruits, vegetables, olive oil and cereals if consumed with each meal; Two points for dairy products and nuts if consumed daily; One point for the recommended number of servings per week is consumed for potatoes (≤3), legumes (≥2), eggs (2–4), fish (≥2), white meat (2), red meat (<2), sweets (≤2) and fermented beverages (1–2 glasses a day) [17].

It sums up to a maximum MDSS score of twenty four (24), and the greater score implies greater adherence to the MeDi. Optimal cut-off point ≥13.50 was set to determine adherence or non-adherence to the MeDi [16]. 

### 2.4. Medical History, Clinical and Laboratory Parameters

By thorough examination of patients medical records, data about existence and duration of primary chronic kidney disease, arterial hypertension, diabetes mellitus, cardiovascular events as well as time of kidney transplantation (KTX), type and duration of dialysis treatment before KTX were obtained. 

Regarding laboratory parameters, all study participants underwent usual peripheral blood sampling, and they were asked to obtain a 24-h urine sample on the same day of the body composition and blood pressure measurement. We collected data on levels of urea (mmol/L), creatinine (mmol/L), uric acid (mmol/L), serum albumin (g/L), phosphates (mmol/L), C-reactive protein (CRP; mg/L), calcium (mmol/L), glucose (mmol/L), triglycerides (mmol/L), total cholesterol (mmol/L), low-density lipoprotein cholesterol (LDL) (mmol/L), haemoglobin (g/L), mean cellular volume (MCV), sodium (mmol/L), potassium (mmol/L), and eGFR using CKD-EPI (mL/min/1.73 m^2^). A complete blood count was obtained using a hematology analyser (Advia 120, Siemens, Erlangen, Germany) and intact parathyroid hormone (PTH; pmol/L) was measured by immunoassay analyzer (Cobas e601, Roche Diagnostics, Penzberg, Germany).

### 2.5. Statistical Analysis

Normal distribution for numerical variables was assessed by the Shapiro-Wilk test. In cases of normal distribution, data was described by mean and standard deviation (SD), while for cases of non-normal distribution, data was described by median and interquartile range (IQR). Categorical variables were described with numbers and percentages. To assess differences between groups, chi-square test was used for categorical variables, *T*-test for parametric numerical variables, and Mann-Whitney U test for non-parametric numerical variables. Finally, univariate and multivariate linear (dependent variable MDSS) and logistic (dependent variable adherence to the MeDi with MDSS cut-off ≥14) regression analyses were performed to examine the effect of other measured parameters with greater adherence to the MeDi. Multivariate models included age, sex, time of kidney transplantation, and duration of dialysis treatment before KTX. In addition, logistic regression analyses were performed separately for each of the 14 foods or food groups to investigate which participants’ characteristics were associated with better adherence to the specific food group. Results of linear regression were provided as beta values (β) with standard errors (SE), while for logistic regression beta values were transformed into odds ratios (OR) with 95% confidence intervals (CI). Significance level was set at *p*-value < 0.05. All statistical analyses were performed using the free software environment for statistical computing R [18].

## 3. Results

One hundred and sixteen (116) KTR patients were enrolled in this study. Table 1 shows the detailed list of demographic, clinical, biochemical and anthropometric parameters of all 116 participants as well as differences between parameters regarding adherence to the MeDi.

Only 29 out of 116 (25%) Dalmatian KTR were adherent to Mediterranean Diet pattern (scored 14 or more on total MDSS score), with mean MDSS score being 10.57. There was no statistically significant difference between the group which is adherent to the MeDi (MDSS ≥ 14) and the group which is non-adherent to the MeDi (MDSS < 14) neither in general parameters regarding medical history, laboratory parameters, anthropometry nor body composition.

### Adherence to MeDi and Its Components

**Figure 1:**
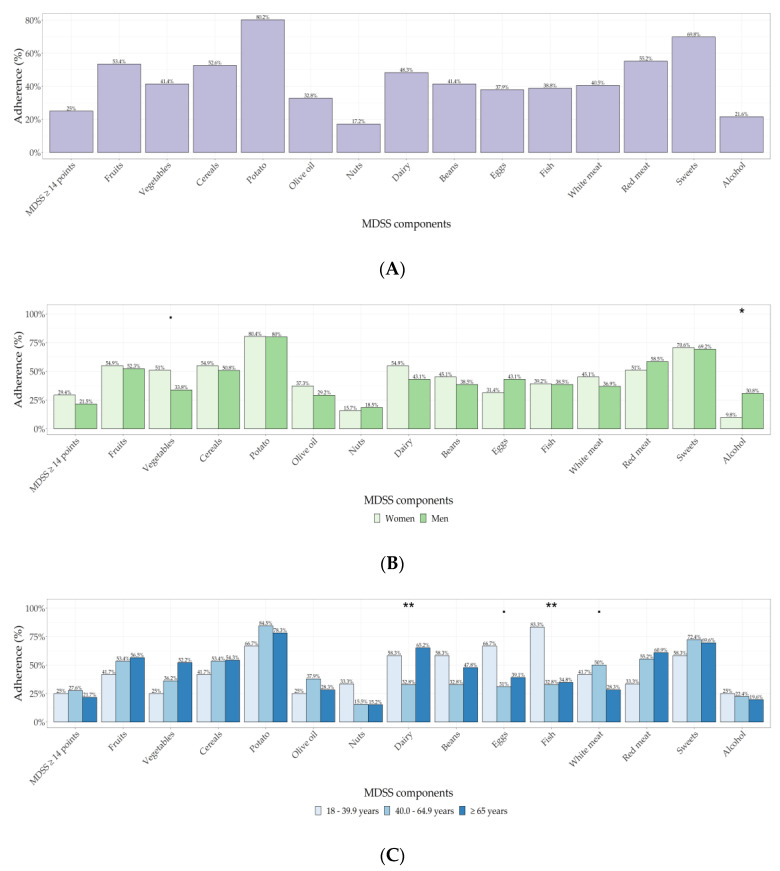
(**A**) Overall adherence to the MeDi and its components. (**B**) Sex differences in adherence to the MeDi. (**C**) MeDi adherence in different age groups of KTR patients. Abbreviations: MeDi—Mediterranean Diet, MDSS—Mediterranean Diet Serving Score, *p*-value < 0.1 depicted with dot, *p*-value < 0.05 depicted with *, *p*-value < 0.01 depicted with **.

When analysing adherence to the recommendations for each food group consumption separately, majority of KTRs followed recommendations on potato consumption (<3 times per week; 80.2%), while 69.8% of KTRs stated that they consume sweets <2 times per week. Only 17.2% KTRs eats nuts daily as MeDi suggests. When it comes to olive oil and fish, only 32.8% of Dalmatian KTRs consume olive oil daily and 38.8% consume fish two or more times per week. Total adherence to the MeDi (MDSS score ≥14 implying good adherence) and adherence to the recommended consumption of food groups is shown in Figure 1A.

Sex differences in adherence to the MeDi and its components are shown in Figure 1B. We found no statistically significant differences in total adherence to the MeDi, and the only statistically significant difference in separate MeDi components was observed for alcohol consumption, where men had higher adherence to the recommendation of alcohol consumption than women (*p* = 0.012).

Adherence to the MeDi and its components in different age groups of KTR patients are shown in Figure 1C. The only statistically significant difference was observed in the recommendations for fish and dairy consumption, where majority (83.3%) of young KTRs (18–40 years old) consume fish >2 times per week, and only 32.8% of middle-aged participants (40–64.9 years old) consume dairy products as recommended by the MeDi.

Differences in MDSS adherence and adherence to its components according to the values of BMI are shown in Figure S1, and no statistically significant difference was found, except for borderline significance for recommendations of sweets consumption, where slightly more KTRs that are obese are following the recommendations (75% vs. around 60% for normal weight and overweight participants).

In addition, when comparing adherence to the MeDi and its components based on the presence of different comorbidities (arterial hypertension (AH), diabetes mellitus (DM), chronic kidney disease (CKD)) we found that adherence to MeDi suggestions of intake of fruits, vegetables and dairy were all higher in KTRs suffering from DM. Full table is shown in Table S1. No other statistically significant difference was observed for other comorbidities.

Statistically significant associations between adherence to the MeDi and measured parameters is shown in Table 2. We found that adherence to the MeDi adjusted for the effects of age, sex, time since transplantation and duration of dialysis treatment prior to the kidney transplantation if expressed as total MDSS score was associated with higher skeletal muscle mass and was higher in participants with DM, while if MeDi was expressed as binary variable (adherent vs. non-adherent participants), we observed significant associations with higher serum levels of albumins and phosphorus.

The complete table of univariate and multivariate linear and logistic regression of MDSS in KTRs can be found in Table S2.

Regression analyses of every food component of MeDi is shown in Table S3. Analyzing adherence to specific food groups in the regression analysis we found an association between better adherence to fruit consumption recommendations and lower phosphorus levels, as well as higher incidence of type 2 DM. Adherence to recommendations of vegetable intake was associated with higher incidence of type 2 DM and higher skeletal muscle mass. There was association between adherence to cereals recommendation and lower levels of potassium, adherence to potato intake recommendations and lower LDL levels. Consumption of olive oil on daily basis was associated with lower triglyceride levels in Dalmatian KTRs.

**Figure 2:**
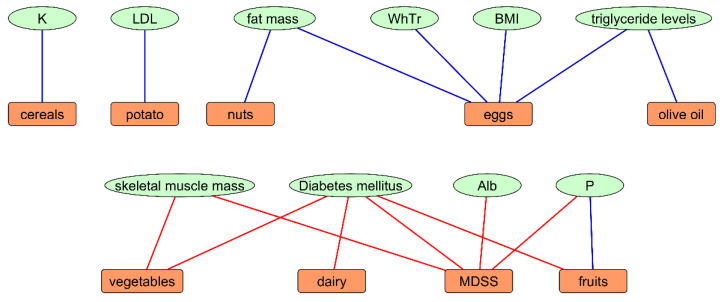
Summary presentation of signifficant associations between adherence to components of the MeDi and researched parameters in Dalmatian KTRs. Explanation: red lines represent positive associations (OR > 1, *p* < 0.05), while blue lines represent negative associations (OR < 1, *p* < 0.05). Abbreviations: MDSS—Mediterranean Diet Serving Score, Alb—serum albumin (g/L), P—phosphates (mmol/L), LDL—low-density lipoprotein cholesterol (mmol/L), K—potassium (mmol/L), BMI—Body Mass Index, WHtR—waist to height ratio, MeDi—Mediterranean Diet.

Daily consumption of nuts was associated with lower levels of fat mass (both in kg and %). Association between following dairy products recommendations and higher incidence of type 2 DM was found. Adherence to MeDi recommendations on eggs intake was associated with lower levels of BMI, WhTr, triglyceride levels, fat mass in percentage (%) and kilograms (kg). A graphical summary of the regression analyses is shown in Figure 2.

## 4. Discussion

In this cross-sectional study, designed to evaluate dietary habits of Dalmatian KTRs, we found very low adherence to the MeDi. To our knowledge, this is the first study that has evaluated MeDi adherence in KTRs in this region. 

Dalmatian kidney transplant recipients showed adherence to the Mediterranean Diet by only 25% despite living in the area where it has been traditionally consumed for centuries and is a part of UNESCO Intangible Cultural Heritage of Humanity [19]. Other research from the same geographical region show the similar adherence to the MeDi (23%) in a healthy population [20] and even lower adherence in patients with inflammatory bowel disease, another chronic non-communicable disease [21]. Other studies with KTRs used a different kind of questionnaire which uses nine food groups to assess adherence to the MeDi. Results of a study from Oste et al. conducted in the Netherlands suggest that 54% of RTRs had higher score on MDS [11], other observational studies did not report a clear cut-off value for adherence to the MeDi [10,14,22,23]. The disheartening number of MeDi-adherent KTRs and the fact that people in other parts of Dalmatia are poorly adherent to the MeDi [20] speak in favour of changes in dietary habits and lifestyle in Dalmatia. Another factor contributing to a low adherence to the MeDi among KTRs can be low levels of food literacy which has been associated with MeDi adherence in KTRs in recent research [23]. These results emphasize the need for nutrition intervention in routine post-transplantation care in Dalmatia.

Our results show no statistically significant difference in laboratory, antropometric nor body mass composition parameters between adherent and non- adherent to the MeDi group of Dalmatian KTRs. The possible explanation of these results can be the small number of KTRs adherent to the MeDi in our study. In contrast to our study, previous study from Oste et al. [11] found lower levels of triglycerides and higher levels of HDL in patients with high MDS. 

When it comes to sex differences, there were no differences in adherence to the MeDi between male and female KTRs. Only significant diffefrence was noticed in higher adherence to alcohol consumption reccomendation in male KTRs. This fact could be explained by that the men in Dalmatia traditionally drink more alcohol, especially wine, than women [24].

No statistically significant difference in adherence to the MeDi according BMI values, neither in MDSS or any food group besides borderline significance for sweets consumption in obese KTRs, shows an overeall poor dietary pattern in participants. Our results showed no differences in adherence to the MeDi in Dalmatian KTRs with present CKD vs. those without. It implies that our patients lack in needed nutritional support and that they are not aware of importance of nutrition being a part of treatment.

The results of our study showed that adherence to the MeDi adjusted for the effects of age, sex, time since transplantation and duration of dialysis treatment prior to the kidney transplantation if expressed as total MDSS score was associated with higher skeletal muscle mass and was higher in participants with DM, while if MeDi was expressed as binary variable (adherent vs. non-adherent participants), we observed significant associations with higher serum levels of albumins and phosphorus.

Our results showed that MDSS is predictor of serum albumin level in KTRs. Higher levels of albumin were associated with better long term posttransplantation outcomes in a study on 404 renal allograft recipients [25]. Our results also suggest that KTRs adherent to the MeDi have a higher absolute value of muscle mass which is also in favor of better nutritional status [22,26].

Significance of following Mediterranean dietary pattern in KTR patients could be in maintaining a good nutritional status and in prevention of sarcopenia and protein energy wasting (PEW) both contributing to high cardiometabolic risk in this specific group of patients [27]. Furthermore, our results suggest associations with MeDi adherence with higher levels of phosphorus. This finding is in contrast to results from Di Iorio et al. [28] who found lower levels of phosphorus in patients suffering from CKD prescribed with MeDi and very-low-protein diet.

These results can be affected by intake of processed food, food additives and preservatives [29] which we did not take into consideration in this study. Higher phosphorus levels in Dalmatian KTRs who are adherent to the MeDi should be monitored and patients should be educated about specific food high in phosphorus and food preservatives so they could avoid complications of hyperphosphatemia [30,31].

Significantly higher MDSS score was found in KTRs with DM. Diabetic KTRs in our study seem to be more aware of the fact that nutrition is the integral part of chronic disease treatment. Furthermore, our results showed higher incidence of DM in those KTRs adhering to MeDi reccomendations on fruit, vegetables and diary products intake.This could be the consequence of eating food with high glycaemic index (GI). Recent meta-analysis states that the added benefit of lower GI version of MeDi in DM prevention [32]. This finding also stresses the importance of personalized nutritional support for this population.

When discussing specific foods and food groups of Mediterranean diet we wanted to highlight the importance of olive oil, fish and nuts consumption in Dalmatian KTRs. Only one third of Dalmatian KTRs consume olive oil daily as MeDi suggests. Also, our results show that those patients who consumed olive oil following the reccomendations of MeDi had statistically lower levels of tryglicerides. Recent findings have highlighted the direct impact of olive oil on HDL levels [33,34], while not having as great impact as other plant oils on lowering the levels of total cholesterol, triglycerides and LDL [34]. PREDIMED study [3] found olive oil supplemented MeDi associated with lower incidence of major cardiovascular events in persons at high cardiovascular risk.

Our results showed that 38% of Dalmatian KTRs consume fish >2 times per week, and most of them are aged between 18–40 years. The possible explanation for this findings could be that younger KTRs are more aware of helathy habits and find themselves in a better financial position, from where they are able to spend more money on specific groceries like fish. Older patients are mostly retired with low household income.

Dalmatian KTRs are the least adherent to nut consumption recommendations where only 17.2% of them consume nuts daily. Although low adherence, it is a lot higher than in general population adherence to nut consumption reported by Relja et al. [35]. Pre-translpalnt food restrictions on avoiding food with high content of potassium and phosphorus may still have residual effect on the dieatry habits in KTRs which could explain this low level of adherence to aforementioned recommendations in this population. The beneficial impact of nuts consumption after KT suggested by our results could be in lower fat mass (both in kg and %). PREDIMED study [3] reported that lower incidence of major cardiovascular events among persons with high cardiovascular risk was in those adherent to MeDi supplemented with nuts. It is well known that obesity and fat mass have been recognized as important factors contributing to high cardiovascular risk in KTRs [8,9].

Regarding other components of the MeDi, fruits, vegetables, cereals, potatoes, eggs and dairy products showed signifficant associations with researched parameters in this population.

Adherence to reccomendations of consumption of fruits with every meal showing association with lower phosphorus levels in Dalmatian KTRs could be explained by the fact that bioavailability of phosphorus from plants is low [29]. Higher incidence of DM in KTRs eating fruits with every meal could be the consequence of eating fruit with high GI. Also, adherence to daily consumption of vegetables as MeDi suggests showed associations with higher muscle mass implying associations between vegetable intake and nutritional status in KTRs.

Furthermore, our results suggest association between lower LDL values and moderate intake of potatoes (three or less times per week) in Dalmatian KTRs. Consumption of cereals with every meal showed an association with lower levels of potassium in our study indicating favourable effects for KTR population. Interestingly, moderate consumption of eggs (2–3 times per week) as suggested by the MeDi showed associations with lower levels of BMI, WhTr, fat mass in kilograms and percentage as well as lower trygliceride levels indicating its potential impact on nutritional status improvement and lowering lipid parameters in KTRs.

There are some limitations to this study. It is a single center, but largest urban center on the Adriatic coast, to where almost 2 million people gravitate. We also included relatively small number of participants in this study but due to the specificity and small number of target population it is a representative sample. Any causal or directional conclusions are halted by the cross-sectional design of the study. In this study we are lacking data on patricipants income, level of physical activity and intake of food additives and perservatives.

## 5. Conclusions

The results of this sudy showed low adherence to the MeDi of Dalmatian KTRs. Surprisingly low adherence is noted for food traditionally and geographically available in this region such as olive oil, fish and nuts. MeDi can have favorable effects on undernutrition and prevention of obesity which is a purpose of nutritional care in post-transplantation period. 

These results point out a necessity of structured nutritional approach in this population of patients and also in different countries. Future studies, with a higher number of participants should be designed to investigate the impact of this intervention on long-term outcomes on nutritional status of KTRs.

## Figures and Tables

**Table 1:** Basic characteristic of study population and differences regarding adherence to the MeDi.

	Total (N = 116)	No Adherence MDSS < 14 (N = 87)	Adherence MDSS ≥ 14 (N = 29)	*p*-Value *
Time since transplantation (years), median (IQR)	4.75 (7)	4.25 (6.62)	5 (8.25)	
	Dialysis type, N (%)			
PD	42 (36.84)	30 (35.29)	12 (41.38)	0.801
HD	65 (57.02)	50 (58.82)	15 (51.72)	
PD + HD	7 (6.14)	5 (5.88)	2 (6.9)	
Dialysis duration (years), median (IQR)	2 (4)	2 (3.67)	2 (3)	0.599
Age (years), median (IQR)	60 (16)	60 (16)	60 (12)	0.833
Sex, N (%)				
Women	51 (43.97)	36 (41.38)	15 (51.72)	0.450
Men	65 (56.03)	51 (58.62)	14 (48.28)	
Arterial hypertension, N (%)	99 (85.34)	73 (83.91)	26 (89.66)	0.649
Diabetes mellitus, N (%)	24 (20.69)	16 (18.39)	8 (27.59)	0.427
Chronic kidney disease (eGFR < 60), N (%)	79 (71.82)	60 (72.29)	19 (70.37)	1.000
**Biochemical parameters**				
Alb (g/L), median (IQR)	42 (4)	42 (4)	43 (5)	0.213
Ca (mmol/L), median (IQR)	2.45 (0.18)	2.45 (0.17)	2.45 (0.19)	0.986
CRP (mg/L), median (IQR)	2.4 (4.15)	2.2 (4.35)	2.9 (3.15)	0.993
E, median (IQR)	4.65 (0.7)	4.62 (0.69)	4.72 (0.57)	0.443
GUP (mmol/L), median (IQR)	5.2 (1.07)	5.2 (1.05)	5.1 (1.05)	0.562
Hb (g/L), median (IQR)	135 (18.5)	133 (17)	136 (18)	0.629
K (mmol/L), mean (SD)	4.12 (0.48)	4.16 (0.47)	3.97 (0.51)	0.065
Total cholesterol (mmol/L), mean (SD)	5.91 (1.3)	5.86 (1.3)	6.05 (1.33)	0.576
Creatinin (mmol/L), median (IQR)	124 (49.75)	127 (47)	115 (86.5)	0.909
LDL (mmol/L), median (IQR)	3.5 (1.48)	3.55 (1.52)	3.1 (1.36)	0.763
MCV (fL), mean (SD)	87.94 (5.52)	87.85 (5.41)	88.25 (5.98)	0.745
Na (mmol/L), median (IQR)	141 (3)	141 (3)	141 (2)	0.852
P (mmol/L), median (IQR)	1.02 (0.24)	1 (0.24)	1.11 (0.27)	0.075
Tgl (mmol/L), median (IQR)	1.8 (1.38)	1.8 (1.2)	1.75 (1.79)	0.894
Uric acid (mmol/L), median (IQR)	394 (86.25)	401 (96.75)	386.5 (66.25)	0.469
Urea (mmol/L), median (IQR)	9.8 (5)	9.6 (4.65)	10.1 (6.4)	0.424
eGFR (mL/min/1.73 m^2^), median (IQR)	47.2 (25.5)	47.1 (24.7)	47.3 (30.5)	0.687
**Anthropometric parameters**				
Height (cm), mean (SD)	173.09 (10.01)	173.34 (9.34)	172.3 (12.06)	0.639
Weight (kg), median (IQR)	77.05 (19.03)	76.4 (19.2)	82.2 (18.4)	0.841
BMI (kg/m^2^), mean (SD)	26.27 (4.01)	26.26 (3.87)	26.3 (4.5)	0.959
BMI (category), N (%)				
<25.0 (normal)	43 (38.39)	33 (38.82)	10 (37.04)	0.836
25.0–29.9 (overweight)	49 (43.75)	36 (42.35)	13 (48.15)	
≥30.0 (obese)	20 (17.86)	16 (18.82)	4 (14.81)	
Middle upper arm circumference (cm), median (IQR)	29 (7)	28 (7)	30 (8)	0.842
Waist circumference (cm), mean (SD)	99.29 (13.03)	99.56 (12.87)	98.59 (13.67)	0.746
WHtR, mean (SD)	0.58 (0.07)	0.58 (0.07)	0.58 (0.08)	0.940
**Body composition parameters**				
Fat mass (kg), median (IQR)	19.1 (10.85)	19.05 (10.95)	19.2 (10.1)	0.775
Fat mass (%), mean (SD)	23.23 (8.87)	23.27 (8.67)	23.1 (9.66)	0.933
Fat free mass (kg), median (IQR)	59.6 (18.35)	59.9 (16.9)	54.7 (21.55)	0.802
Visceral fat, mean (SD)	9.13 (3.85)	9.18 (3.82)	8.96 (4.02)	0.803
Muscle mass (kg), median (IQR)	56.6 (17.8)	56.9 (16.75)	51.9 (20.55)	0.821
Skeletal muscle mass (kg), median (IQR)	32 (12.3)	32.25 (11.48)	27.2 (16.2)	0.799
Skeletal muscle mass (%), median (IQR)	41.4 (8.85)	41.15 (8.5)	43.5 (10.35)	0.834
Body mass (kg), median (IQR)	3 (0.8)	3 (0.72)	2.8 (1)	0.744
Trunk visceral fat (kg), median (IQR)	10.1 (6.75)	9.9 (6.58)	10.45 (7.65)	0.994
Total MDSS points, mean (SD)	10.57 (4.15)			
MDSS ≥ 14 points, N (%)	29 (25)			

Abbreviations: PD—peritoneal dialysis, HD—hemodialysis, MDSS—Mediterranean Diet Serving Score, Alb—serum albumin (g/L), P—phosphates (mmol/L), C-reactive protein (CRP; mg/L), Ca—calcium (mmol/L), GUP—glucose (mmol/L), triglycerides (mmol/L), LDL—low-density lipoprotein cholesterol (mmol/L), Hb—haemoglobin (g/L), MCV—mean cellular volume, Na—sodium (mmol/L), K—potassium (mmol/L), eGFR—estimated glomerular filtration rate using CKD-EPI (ml/min/1.73 m^2^), E—eritthrocyte count, BMI—Body Mass Index, WHtR—waist to height ratio. * *p*-values were obtained with chi-square test for categorical variables, *T*-test for parametric numerical variables, and Mann-Whitney U test for non-parametric numerical variables.

**Table 2:** Association between adherence to the MeDi and measured parameters (only statistically significant values shown).

Predictor	MDSS Score (Linear Regression)			MDSS Cut Off 14 Points (Logistic Regression)		
	Adjusted for Age, Sex, Time Since KTX, Dialysis Duration			Adjusted for Age, Sex, Time Since KTX, Dialysis Duration		
	Beta	SE	P	OR	95% CI	P
Diabetes mellitus	2.545	1.198	0.037	2.22	0.6–8.19	0.232
Alb (g/L)	0.106	0.114	0.356	1.19	1–1.41	0.046
P (mmol/L)	2.909	2.130	0.176	13.36	1.16–154.15	0.038
Skeletal muscle mass (kg)	0.240	0.102	0.021	1.09	0.96–1.23	0.184

Abbreviations: MDSS—Mediterranean Diet Serving Score, Alb—albumin level (g/L), P—phosphates (mmol/L), KTX—kidney transplantation, MeDi—Mediterranean diet, CI—confidence interval, SE—standard error, OR—odds ratio.

## Data Availability

Raw data can be found at corresponding author via e-mail: josiparadic1973@gmail.com.

## Supplementary Materials

The following are available online at https://www.mdpi.com/article/10.3390/nu13093246/s1, Figure S1: Adherence to the MeDi according to values of BMI in Dalmatian KTRs, Table S1: Differences in adherence to Mediterranean diet and its individual components based on patients’ comorbidities, Table S2: Regression analysis of MDSS in Dalmatian KTRs, Table S3: Regression analysis of components of MDSS in Dalmatian KTRs.
