# Supplementary material for: Mediterranean Diet Adherence and Nutritional Status in Dalmatian Kidney Transplant Recipients—Are They Related?
Source: Nutrients. 2021 Sep 18;13(9):3246. doi: 10.3390/nu13093246 (PMC8471203; doi:10.3390/nu13093246)
Supplement: Supplementary file 1 [file nutrients-13-03246-s001.zip › Supplementary Figure S1 and Supplementary Table S1 - AG30082021.pdf]

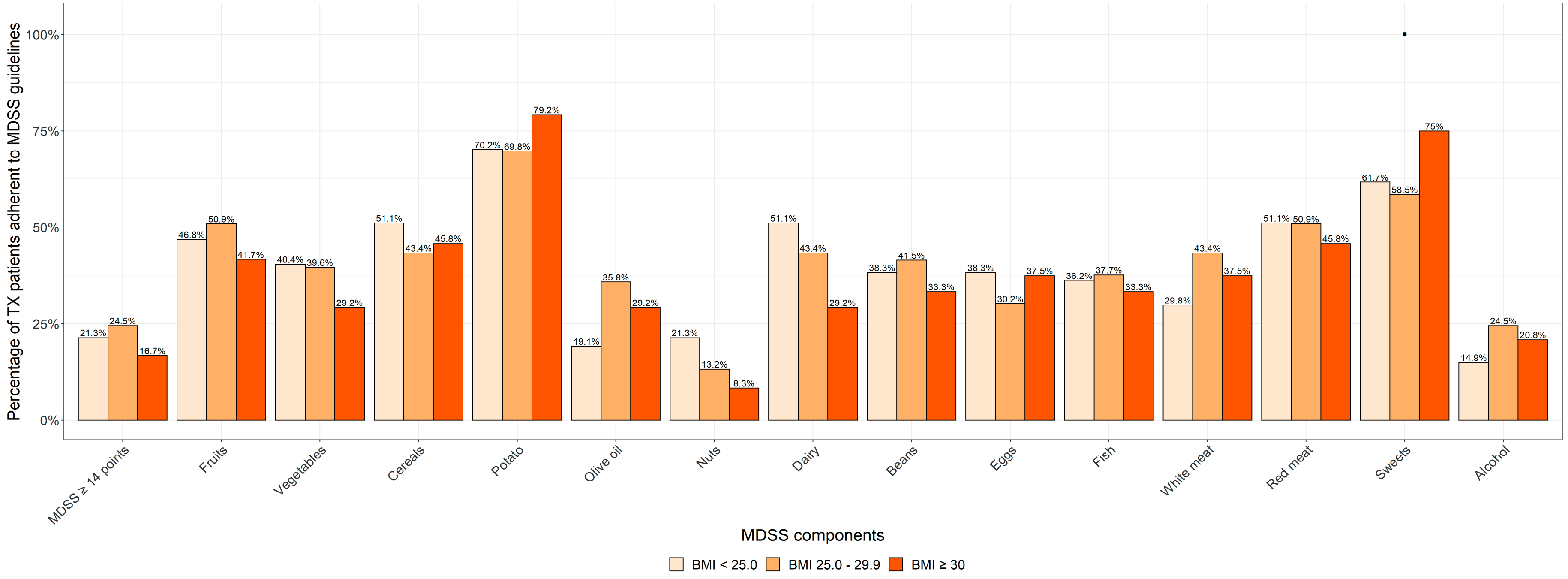

Supplementary Figure S1. Adherence to the MeDi according values of BMI in Dalmatian KTRs

Abbreviations: MDSS Mediterrranean Diet Serving Score, MeDi - Mediterranean diet, KTR - kidney transplant recipient, BMI - Body Mass Index

Supplementary Table S1. Differences in adherence to Mediterranean diet and its individual components based on patients comorbidities

	Arterial hypertension			Diabetes mellitus			Chronic kidney disease (eGFR < 60ml/min)		
	**No (N = 17)**	**Yes (N = 99)**	***p*-value***	**No (N = 92)**	**Yes (N = 24)**	***p*-value***	**No (N = 31)**	**Yes (N = 79)**	***p*-value***
**Total MDSS points**, mean (SD)	10.59 (4.65)	10.57 (4.09)	0.984	10.07 (4.16)	12.5 (3.56)	**0.010**	10.68 (3.9)	10.44 (4.08)	0.784
**MDSS ≥ 14 points**, N (%)	3 (17.65)	26 (26.26)	0.649	21 (22.83)	8 (33.33)	0.427	8 (25.81)	19 (24.05)	1.000
**Fruits**, N (%)	9 (52.94)	53 (53.54)	1.000	44 (47.83)	18 (75)	**0.032**	18 (58.06)	41 (51.9)	0.711
**Vegetables**, N (%)	5 (29.41)	43 (43.43)	0.413	31 (33.7)	17 (70.83)	**0.002**	14 (45.16)	31 (39.24)	0.724
**Cereals**, N (%)	9 (52.94)	52 (52.53)	1.000	48 (52.17)	13 (54.17)	1.000	14 (45.16)	44 (55.7)	0.433
**Potato**, N (%)	14 (82.35)	79 (79.8)	1.000	75 (81.52)	18 (75)	0.670	28 (90.32)	62 (78.48)	0.240
**Olive oil**, N (%)	5 (29.41)	33 (33.33)	0.969	31 (33.7)	7 (29.17)	0.860	11 (35.48)	24 (30.38)	0.772
**Nuts**, N (%)	5 (29.41)	15 (15.15)	0.276	16 (17.39)	4 (16.67)	1.000	7 (22.58)	11 (13.92)	0.414
**Dairy**, N (%)	10 (58.82)	46 (46.46)	0.497	39 (42.39)	17 (70.83)	**0.024**	14 (45.16)	38 (48.1)	0.948
**Beans**, N (%)	9 (52.94)	39 (39.39)	0.435	38 (41.3)	10 (41.67)	1.000	11 (35.48)	34 (43.04)	0.610
**Eggs**, N (%)	6 (35.29)	38 (38.38)	1.000	36 (39.13)	8 (33.33)	0.776	11 (35.48)	31 (39.24)	0.883
**Fish**, N (%)	6 (35.29)	39 (39.39)	0.959	38 (41.3)	7 (29.17)	0.394	15 (48.39)	27 (34.18)	0.245
**White meat**, N (%)	5 (29.41)	42 (42.42)	0.458	35 (38.04)	12 (50)	0.407	12 (38.71)	33 (41.77)	0.938
**Red meat**, N (%)	10 (58.82)	54 (54.55)	0.949	47 (51.09)	17 (70.83)	0.133	16 (51.61)	44 (55.7)	0.862
**Sweets**, N (%)	14 (82.35)	67 (67.68)	0.351	62 (67.39)	19 (79.17)	0.385	20 (64.52)	57 (72.15)	0.579
**Alcohol**, N (%)	2 (11.76)	23 (23.23)	0.457	23 (25)	2 (8.33)	0.136	5 (16.13)	19 (24.05)	0.517

* *p*-values were obtained with chi-square test for categorical variables, and T-test for parametric numerical variables

MDSS- Mediterranean Diet Serving Score

eGFR- estimated flomerular filtration rate
